# Online teaching during the COVID-19 pandemic: exploring science/STEM teachers’ curriculum and assessment practices in Canada

**DOI:** 10.1186/s43031-022-00048-z

**Published:** 2022-03-07

**Authors:** Isha DeCoito, Mohammed Estaiteyeh

**Affiliations:** Faculty of Education, Western University, 1137 Western Road, London, ON N6G 1G7 Canada

**Keywords:** Emergency remote teaching, Online teaching, Science/STEM education, Curriculum, Assessment, TPACK, Student outcomes

## Abstract

The COVID-19 pandemic necessitated school closures globally, resulting in an abrupt move to online/distance teaching or emergency remote teaching (ERT). Teachers and students pivoted from face-to-face engagement to online environments, thus impacting curriculum, pedagogy, and student outcomes across a variety of disciplines. In this paper, the authors focus on science/STEM teachers’ experiences with online teaching and learning in a Canadian context during the pandemic. Qualitative and quantitative data were collected through an online questionnaire administered to 75 Grade 1–12 science/STEM teachers in a Canadian province in May–July 2020. Through the TPACK framework and self-efficacy theory, the authors explore i) curriculum planning and implementation in online settings, ii) assessment practices and their effectiveness, and iii) student outcomes, as observed by the teachers. Results indicate that teachers used a variety of platforms, and choice of platform was mainly due to user-friendliness and interactivity, or administrative decision making. Despite teachers organizing online lessons during ERT, gaps were identified in teachers’ TPACK framework and self-efficacy, thus impacting their curriculum development, pedagogical approaches, and assessment practices. In general, teaching strategies included pre-recorded videos and self-directed learning in which teachers assigned specific tasks for students to perform independently. Teachers prioritized subject content and covering curriculum objectives over creative and student-centered pedagogical approaches. Assessment techniques employed were viewed by teachers as unauthentic and generally ineffective. Moreover, teachers reported difficulties addressing student needs and abilities, resulting in challenges providing equitable and inclusive online teaching. Finally, online teaching was viewed negatively by most teachers, in terms of student engagement and outcomes.

## Introduction

Due to the COVID-19 pandemic, around 1.7 billion students were impacted by school closures in 190 countries in 2020 and 2021 (Barron Rodriguez et al., 2021). Various countries reacted differently to their educational systems’ interruption. Some countries and schools opted to rebalance the curriculum by prioritizing core content deemed essential for student progression and examinations, often focusing on literacy and numeracy (Reimers & Schleicher, 2020). Meanwhile, technology was undoubtedly an indispensable tool in remote learning. For instance, in China nearly 200 million primary and secondary school students learned online. This was considered the largest simultaneous online learning exercise in human history. Additionally, the UAE government offered free digital education to 50 million Arab schoolchildren (Barron Rodriguez et al., 2021).

Countries used educational technology to various extents (e.g., computers, radio, television, social media) to enable access to remote learning during the pandemic. For example, Bangladesh broadcasted lessons for students in grades six to ten on national television​. These lessons were also accessed as on-demand content on YouTube. In Afghanistan, distance learning combined with multimedia was initiated, but later aborted after facing challenges due to insufficiencies with the existing infrastructure. In Rwanda, the Rwanda Education Board broadcasted education radio programs on its district-level radio stations to support students with remote learning (Barron Rodriguez et al., 2021), similar to the Australian Outback model of learning “School of the Air”.

Certain countries, such as Finland, were better prepared to switch to an exclusively virtual learning environment as they were already widely in use before the pandemic. Online digital platforms were used for student assignments, assessments, and home-school communication. Moreover, teachers and students had access to a content repository that incorporated resources and applications to facilitate online education. Accordingly, relatively minimal disruptions were noted. Similarly, in Japan, a centralized link was used to synthesize all information related to the COVID-19 response undertaken by schools. Coping strategies included technology-based distance learning (e.g., online-class delivery and videoconferences), as well as maximizing the use of school grounds and facilities safely (Barron Rodriguez et al., 2021).

In Canada, 6,643,213 students were affected by school closures (UNESCO, 2020). Provincial and territorial ministries of education instructed schools to migrate to online/distance teaching for all K-12 students. Some provinces instructed that elementary teachers focus on mathematics and literacy, while secondary teachers focus on literacy, math, and sciences, with a notable emphasis on science, technology, engineering and mathematics (STEM) subjects. Teachers were provided minimal suggestions for resources and platform usage during this process.

Hodges et al. (2020) maintain that well-planned online learning experiences differ from online courses offered in response to a crisis or disaster and define emergency remote teaching (ERT) “as a temporary shift of instructional delivery to an alternate delivery mode due to crisis circumstances” (p. 6). Hodges et al. (2020) state that effective online learning and quality online teaching require careful instructional design, planning, and development, as well as an investment in the support systems. These conditions are mostly absent in emergency shifts, which may reduce the quality of online courses in emergency situations such as the COVID-19 pandemic.

The abrupt world-wide shift to online teaching was accompanied by many challenges. Almost all pedagogical approaches, subject content areas, lesson pacing, interaction models, and assessment methods were modified during the transition. This increased the burden on teachers who were required to align digital educational content with their existing national curricula and concurrently cater for students’ academic, mental health, social, and emotional needs (Barron Rodriguez et al., 2021). In the literature, documented exemplary online teaching approaches are rare because it requires the effective integration of pedagogy, technology, and subject content often referred to as technology, pedagogy, and content knowledge (TPACK) (Koehler & Mishra, 2009), expanded upon in the theoretical framework section. Furthermore, the literature on teachers’ readiness to integrate technology into STEM practice is limited (DeCoito, 2020; DeCoito & Richardson, 2018a, 2018b, 2020a; Reimers & Schleicher, 2020). The aforementioned challenges and gaps warrant exploring teachers’ online practices during ERT.

Since online teaching was a novel experience for many educators, it is crucial to explore how science/STEM teachers functioned in this new teaching and learning environment. Literature on online teaching in K-12 settings is sparse, especially in a Canadian context (Barbour, 2018; Taie et al., 2019; Tallent-Runnels et al., 2006). The authors contend that there is a dire need to explore the online teaching and learning experiences from process and outcome perspectives, including teaching and assessment practices, and learning outcomes. Thus, the overall study focused on science/STEM teachers’ online practices during the pandemic in Canada. In this paper, the authors explore i) curriculum planning and implementation in online settings, ii) assessment practices and their effectiveness, and iii) student outcomes, as observed by the teachers. Specifically, the following research questions were explored: 1) What digital tools and resources were teachers using in an online environment? 2) What strategies did teachers’ online curriculum development and implementation embrace? 3) What models of student assessment did teachers implement online? and 4) What were the impacts of online teaching on students’ outcomes, as observed by teachers?

## Literature review

### Online teaching

Online teaching, also referred to as virtual learning, cyber learning, and e-Learning is the form of learning where individuals are not physically present in a classroom, and where instruction and content are conveyed primarily over the Internet (Schwirzke et al., 2018; Thoms & Eryilmaz, 2014). Online learning has become more prevalent in formal post-secondary education settings in which interactive telecommunications platforms are utilized to connect learners, resources, and instructors (Simonson, 2003, as cited in Simonson et al., 2009). In fully online K-12 school programs, referred to as cyber-schools, students are enrolled primarily (often only) in online classes, and are certified by earning the required credit and diplomas (Schwirzke et al., 2018).

Due to the advancements of technology and its flexibility and ubiquity, online teaching/ learning has evolved over the last 20 years. This has led to its adoption by many higher education institutions around the world, and to a less but growing extent in the K-12 educational system (Barbour, 2018). Nevertheless, the enrollment in K-12 online learning is growing rapidly. In the United States for example, in 2000–2001, 90% of public 2-year and 89% of public 4-year institutions offered distance education courses, accounting for an estimated 2.9 million students enrolled in online programs (Tallent-Runnels et al., 2006). By 2012, all 50 states were offering K-12 online learning opportunities. During the 2017–18 school year, 21% of public and 13% of private schools in the USA offered courses entirely online (Taie et al., 2019). In Canada, online teaching is more dominant in higher education institutions than in K-12 educational systems. The *State of the nation study: K-12 e-learning in Canada* reports that in the Canadian province of Ontario, for example, there were 81 distance education programs for K-12 in 2018 (Barbour & LaBonte, 2019). By comparison, in the same year there were 981 fully online certificate, diploma and degree programs, and more than 20,000 online courses offered to post-secondary (college and university) students (Contact North, 2019). Given the increase in online adoption at the K-12 level, the province of Ontario initiated mandatory e-Learning as a requirement for high school graduation (Barbour & LaBonte, 2019).

### Online teaching affordances and challenges

Online teaching offers many affordances over traditional teaching methods, including enhancing students’ motivation, interaction, and communication (Amasha et al., 2018; Thoms & Eryilmaz, 2014). Student motivation in an online setting is linked to students’ active engagement with learning, enjoyment of learning, perceptions of learning, learner satisfaction, online participation, and academic performance (Hartnett, 2018; Simonson et al., 2009). Simonson et al. (2009) noted that distance education can be as effective as traditional teaching in terms of learner outcomes, and that online learners have more favorable attitudes towards learning than traditional learners. Online courses also provide a distinct advantage for some learners, especially shy students (Smith et al., 2018).

These affordances are also accompanied by challenges and obstacles. While synchronous platforms offer opportunities for interactivity and relationship-building (Smith et al., 2018), it is widely believed that the greatest disadvantage of online learning is its isolating and impersonal nature (Searls, 2012). Students learning online tend to have less opportunities to interact with teachers and peers, which in turn may affect their motivational, cognitive, and affective outcomes (Zhang & Lin, 2020). Additional barriers include i) inadequate technology access, ii) lack of equipment and infrastructure, iii) teachers’ time management due to increased workload, iv) teachers’ and students’ technological skills, v) teachers’ self-efficacy in navigating online environments, and vi) lack of or ineffective teacher training (Barril, 2018; Ferri et al., 2020; Recker et al., 2013; Simonson et al., 2009; Tinoca & Oliveira, 2013).

It is worth noting that many teachers find certain academic subjects and in-school activities resistant to effective transfer to an online learning environment without significant modifications/accommodations (Barron Rodriguez et al., 2021). Teachers face challenges in nurturing higher order thinking and implementing student-centered teaching methods in online classrooms (Baran et al., 2011). This resonates with many science/STEM teachers who find it challenging to perform hands-on inquiry activities and lab-based experiments and are challenged to demonstrate STEM concepts in a virtual environment. These challenges reiterate the need to explore science/STEM teachers’ online practices during ERT.

### Curriculum, teaching, and learning in online environments

Online resources have the potential to enrich classroom environments and promote student learning (Recker et al., 2013) by providing high-quality and collaborative online learning experiences (Hoffman, 2018). These digital tools must ensure students’ self-construction of knowledge, situating learning within real-world circumstances, and creating learning communities that favor student interaction. To utilize these tools effectively, both in-service and pre-service teachers require mastery of digital literacy skills (Ng, 2013). Digital literacy includes digital competence (skills, concepts, approaches, attitudes), digital usage (professional/discipline application), and digital transformation (innovation/creativity) (Belshaw, 2012). Mastering digital skills also necessitate new skills in synchronous and asynchronous online lesson planning and implementation. Thus, online environments demand teacher competence in course design and selecting appropriate curricular materials (Recker et al., 2013); pedagogical approaches (King, 2002; Simonson et al., 2009); and communication techniques (Fernández et al., 2017).

Designing high-quality online courses can be challenging. Lee et al. (2004) advocate for learner-centered digital content, and for humanizing e-Learning by adding more interactivity through media-rich and engaged learning, and collaborative community learning. Criteria identified as necessary for successful online courses include accessibility of the course content, positive learner experience, active engagement and collaboration with peers, work ethics, course design and relevance, diversity in content and activities, accommodating to the many different learner needs, and a balance between formative and summative learning assessments (Leire et al., 2016).

It is important that teachers combine several different learning activities to address a variety of learners’ needs, enhance connectivism (Dipietro, 2010; Leire et al., 2016), and accommodate their different learning styles (Dipietro et al., 2008; Smith et al., 2018). Offering the same material in different forms (such as forum discussions, seminars, and video lectures) allows for flexibility and enhances the understanding of complex topics. Lister (2014) sums up four main considerations when designing online courses, including i) establishing clearly communicated expectations, rubrics, assignments incorporating authentic tasks that include real-life activities, and promoting active learning through reflection and self-assessments, ii) developing content that engages learners, iii) fostering collaboration and interaction, and iv) providing timely feedback.

Teaching practices are key to online learning success and are also a significant predictor of students’ learning perceptions and satisfaction. Unfortunately, limited research has documented practices related to teaching and learning online (Baran et al., 2011; Dipietro et al., 2008). Baran et al. (2011) documented online teachers’ tendency to maintain traditional teaching practices in an online environment due to the quality of the learning management system (LMS). The top-down learning experiences and the directive approach in most LMSs encourage and reinforce direct lecturing and traditional assessment practices (Loertscher & Koechlin, 2013). It is therefore important to place students at the center of the learning experience, and not the technology (Dipietro, 2010), given student participation and engagement are key to successful online teaching (Smith et al., 2018; Vivolo, 2019). This requires an extensive knowledge of how students learn in order to scaffold their learning, with more emphasis on discussions, group projects, effective questioning, and learning communities (Boettcher & Conrad, 2021; Vivolo, 2019). Moreover, some strategies for promoting motivation in online education include inclusivity, developing students’ attitudes, enhancing meaning by highlighting learners’ perspectives, and engendering student competence (Hartnett, 2018). Smith et al. (2018) also recommend additional practical measures to enhance student engagement including constant polling; sharing multimedia; digital storytelling; video journaling; use of interactive tools; recordings, videos, and screen-casting; podcasting; presentations; and virtual reality experiences.

The online environment is conducive to building learning communities in which diverse learners work together in a way that matches contemporary education and workplace settings (Barril, 2018). This requires humanizing digital pedagogy and promoting equity and collaborative learning through dialogue and discussions (Palloff & Pratt, 2007). Barril (2018) also promotes integrating collaborative inquiry-based approaches encompassing problem-based, case-based (DeCoito & Fazio, 2017), and project-based learning methods (DeCoito, 2015), which have been effectively implemented in online learning environments. Through these methods, teachers can incorporate authentic, relevant, real-world questions and problems, whereby they assume the role of facilitator. In the context of science/STEM education, research has shown the efficacy of digital web-based tools for engaging learners and promoting inquiry-based STEM learning (DeCoito & Richardson, 2018a). Equally important, the integration of digital tools has the potential to enhance students’ scientific literacy (Ng, 2013). Specifically, science/STEM teachers have at their disposal a wide variety of digital tools including virtual laboratories (Gröber et al., 2007), computer simulations (Domínguez et al., 2013; Finkelstein et al., 2005), digital video games (DeCoito & Briona, 2020), and digital timelines (DeCoito, 2020). Additional tools include Internet-supported student research projects, modelling software, digital stories, data-logging for data collection, text and multimedia-editing software, and collaborative online environments (Ng, 2013). Through these tools, students can visualize processes better, collect data, organize material for particular purposes such as graphing, and displaying concepts in multiple representations (Ng, 2013; Webb, 2005). These tools highlight how online technologies can be utilized effectively in science/STEM classrooms if teachers are trained and well-prepared to implement them.

### Assessment in online teaching

Online environments are conducive for developing collaborative and creative authentic assessments (McVey, 2016). Nevertheless, several studies have highlighted challenging aspects of assessment, especially homework and formative assessment in online teaching (Amasha et al., 2018; Eichler & Peeples, 2013; Espasa & Meneses, 2010; Tinoca & Oliveira, 2013). Anderson (1998) links teachers’ choice of assessment strategies to their centeredness of teaching, thus teaching and assessment components are highly interrelated. More recently, Pereira et al. (2010, as cited in Tinoca & Oliveira, 2013) proposed a conceptual framework for assessment in an online environment based on four dimensions: authenticity, consistency, transparency, and practicality.

To assess student online learning effectively, teachers are encouraged to use regular, ongoing, and multiple assessment strategies (Dipietro et al., 2008). Furthermore, online assessment practices must also attend to student diversity and inclusion (Barril, 2018; Loertscher & Koechlin, 2013). Online assessment strategies include interactive quizzes, activities, and innovative assessments that can reduce boredom and increase interaction with course material (Smith et al., 2018) such as projects, portfolios, self-assessments, peer evaluations, and immediate feedback (Gaytan & McEwen, 2007).

Of equal importance is the use of timely and corrective feedback facilitated by digital platforms utilized in online teaching (Dipietro, 2010; Vonderwell et al., 2007). These platforms enable formative assessment by collecting student evidence and providing them with feedback. Moreover, timely feedback is associated with higher levels of student performance and satisfaction (Espasa & Meneses, 2010). This is crucial for students to achieve their learning goals, and for teachers to reflect on their online practices (Faber et al., 2017).

Finally, assessment of personal, inter-personal, and high order thinking skills in online classes warrants special mention. Teachers can evaluate communication skills, collaboration, and active learning if their teaching methods facilitate active participation and student engagement. Assessing group work requires attending to a variety of collaboration processes (i.e., interaction, negotiation, knowledge construction), and to individual participation and contribution (Barril, 2018). For example, critical-thinking skills can be assessed by analyzing discussion postings, as well as peer assessments and individual reflections (Hamann et al., 2016; Leire et al., 2016).

Developing technologies are also promising in terms of facilitating assessment and alleviating challenges. According to Eichler and Peeples (2013), adaptive learning systems, generally defined as technology that provide immediate feedback to learners during a learning activity, have proven to enhance student performance. In addition, the authors note that adaptive systems have the potential to differentiate instruction by adjusting the pace and levels of difficulty of the assigned work for both advanced and challenged learners. In contrast, responsive systems provide feedback, tutorials, or hints to learners and engage them in the same set of exercises, in the same order and at the same pace. When compared to traditional methods of assessment, responsive systems are preferred by both student and teacher. Other technology advancements, such as learning analytics, are being used to predict when learners are struggling (Vivolo, 2019). Another promising tool (Kuosa et al., 2016) uses data mining techniques and interactive visualizations to automatically analyze students’ activity and work (such as time spent, chapters read, etc.). Such tools are good examples of “assessment for learning” and “assessment as learning” and can assist students in improving their self-regulation. These tools can also support teachers in evaluating students’ learning performance and monitoring their progress during courses, and support decisions regarding their pedagogical strategies and instructional guidance.

## Theoretical frameworks

**Fig. 1:**
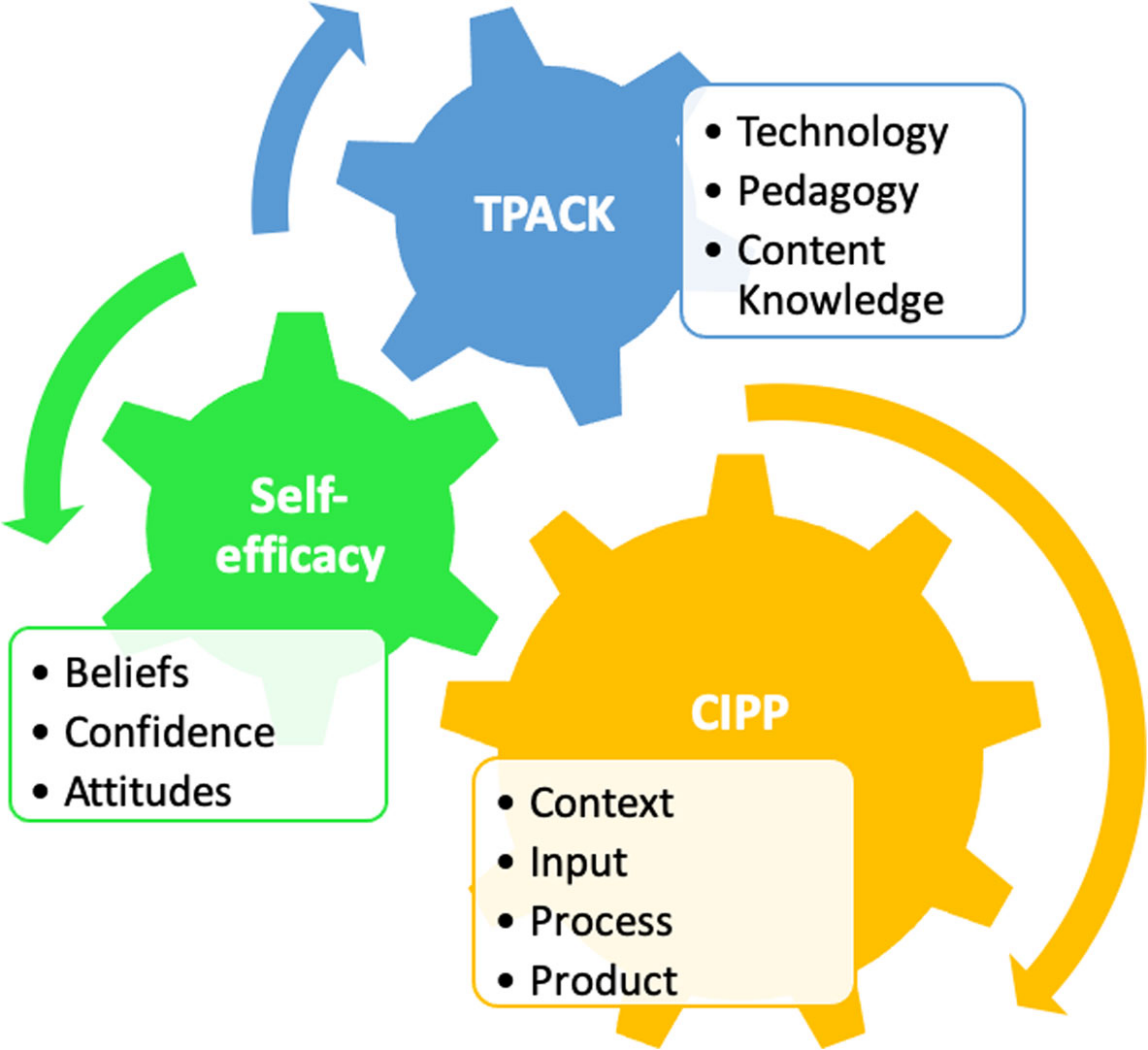
The integration of self-efficacy, TPACK and the CIPP model

In the absence of ERT, online teaching can be evaluated using several frameworks. However, during crisis states, the context, input, process and product (CIPP) model is an appropriate framework for evaluating online teaching (Stufflebeam & Zhang, 2017). Effective science/STEM teaching requires proficiency in science/STEM pedagogical content knowledge (Shulman, 1986) as well as increased self-efficacy in teaching said content (Tschannen-Moran & Hoy, 2001). Additionally, knowledge of TPACK and effective implementation strategies (Koehler & Mishra, 2009) are paramount for successful online teaching. Aspects of the CIPP model and the aforementioned theories informed our theoretical framework as they complement each other and are suitable to predict and interpret science/STEM teachers’ online practices (Fig. 1).

### CIPP model

The CIPP evaluation model is a comprehensive framework utilized in evaluating programs, projects, products, institutions, and systems (Stufflebeam, 2007). The CIPP is based on a systematic approach that entails four components: 1) the context (needs, problems, assets, and opportunities); 2) the inputs (strategy, action plan, staffing arrangements, and budget); 3) the process (monitoring, documenting, assessing, and reporting on the implementation of plans); and 4) the products (costs and outcomes) (Stufflebeam & Zhang, 2017). In this paper, the authors focus on the context (online learning via ERT during the COVID-19 pandemic, teacher and student needs, access and availability of digital tools), the process (curriculum and digital resources, teaching and assessment using digital tools), and the products (teacher reflections and student outcomes).

### TPACK

Good online teaching requires a transactional relationship and complex interaction between all three components suggested by the TPACK framework: technology, pedagogy, and content knowledge (Archambault & Crippen, 2009; Ng, 2013). Koehler and Mishra (2005) explain the TPACK framework as the interactions between the three types of knowledge: technological knowledge (TK), content knowledge (CK), and pedagogical knowledge (PK). TK refers to knowledge about technologies used in teaching and learning. It comprises the digital literacy skills whereby teachers use technology to support pedagogy. PK entails the methods and strategies of teaching and learning. It includes integrating relevant and authentic tools and strategies to engage students in conceptualizing concepts. Finally, CK refers to the subject area understandings and being aware of students’ preconceptions and misconceptions (Koehler & Mishra, 2009; Ng, 2013; Pringle et al., 2015).

Ng (2013) reports on the lack of quality use of information and communications technology in teacher education, as well as a lack of confidence in using technology for preservice teachers, and maintains that the more digitally literate the teacher, the more confidently they will use digital technology in their teaching. For example, studies of digital scientific timelines (DeCoito, 2020), digital video game development (DeCoito & Briona, 2020), and STEM projects (DeCoito, 2015) demonstrated teacher candidates’ ability to translate and integrate TPACK into their curriculum development and future practices. This reiterates the importance of teacher preparation – pre-service and in-service – to enable teachers to effectively integrate technology in their teaching through mastering the TPACK components and connections, thus enhancing their readiness for online teaching.

### Self-efficacy theory

Bandura (2006) defines self-efficacy as “people’s beliefs in their capabilities to produce given attainments” (p. 307). Thus, teachers’ beliefs greatly affect their own teaching practices (E. A. Davis et al., 2006). These beliefs impact the learning environments teachers create for their students and hence affect students’ motivation and learning levels (Bandura, 1993). Similarly, when it comes to integrating technology in teaching, teachers’ technological self-efficacy is the strongest indicator of technology use in their practice (Chen, 2010). This justifies why teachers who are skeptical about instructional technologies may not experiment with new technologies (Fernández et al., 2017). Bandura (1993) maintains that self-efficacy influences cognitive development through four processes: cognitive, affective, motivational, and selection. These four processes take into consideration personal factors, interactions with peers, feedback received, and the level of control over the self and the environment. Therefore, the importance of the affective and the motivational components is significant, since any deficits related to these factors may cause anxiety that would negatively affect performance.

## Methodology

### Research design

Due to the social distancing measures implemented, online questionnaires were the most convenient means of data collection in the setting of a large Canadian province. This study utilizes a mixed-methods design (Creswell & Creswell, 2018) to address the research questions. Quantitative and qualitative data were collected from teachers through an online questionnaire. Our aim was to obtain both comprehensive quantitative and rich qualitative data detailing teachers' experiences with online teaching. To maintain participant anonymity, the questionnaires were de-identified at administration. As teachers were under significant stress and increased workloads during data collection (May–July 2020), the authors elected to refrain from conducting participant interviews.

Participant recruitment methods included snowball sampling through teacher networking and referral (Parker et al., 2019). Teachers were invited to participate in the study through email from school boards and teacher associations. In addition, researchers and consenting teachers recruited additional participants via social media (e.g., Twitter, Facebook, LinkedIn).

### Participants

The questionnaire was administered to science/STEM teachers (*n* = 75) from different locations in a Canadian province. Participants represented various demographics in terms of the grades taught (elementary and secondary), age groups, teaching experience, and education. Participants included science/STEM subject teachers (biology, chemistry, environmental sciences, physics, earth sciences, general science, technology, and mathematics). Their educational background included those with a bachelor’s degree (71%), and graduate degrees (masters or doctorate/29%). Seventy-two percent of respondents were between 31 and 50 years, 16% between 21 and 30 years, and 12% over 50 years of age. Teaching experience varied, with 15% of the respondents having less than 5 years teaching experience. Eighty-five percent had over 5 years teaching experience, while 24% reported 6–10 years teaching experience, 38.5% reported having 11–20 years experience teaching, and 22.5% having more than 20 years teaching experience. Finally, 51% of the participants taught elementary and middle-school (grades 1–8), while the remainder taught secondary school (grades 9–12). Table 1 details the distribution of teachers’ demographics in relation to age ranges. It is worth noting that while the majority of teachers in our study have a bachelor’s degree, most of the teachers with a graduate degree are between 31 and 50 years of age and possess 6–20 years of teaching experience. Moreover, results of the Spearman correlation illustrate a strong positive correlation between teachers’ age and their teaching experience (rs = .65, *p* < .01).

**Table 1:** Details of teachers’ demographics: distribution of teachers within each age range

Age (years)	Sample (n)	Grades taught	Educational background		Teaching experience (years)				
		Gr. 1–8	Gr. 9–12	Bachelor’s	Graduate	1–5	6–10	11–20	20+
21–30	12	6	6	9	3	9	3	0	0
31–40	19	12	7	13	6	0	10	9	0
41–50	35	18	16	24	10	2	4	16	13
51–60	7	2	5	5	2	0	1	4	2
61+	2	0	2	1	0	0	0	0	2
Total	75	38	36	52	21	11	18	29	17

*Note:* One teacher did not indicate their class taught, and two teachers did not indicate their educational background

### Data sources

Participants completed a 5-point Likert scale questionnaire consisting of 24 statements and five open-ended questions. Questionnaire items were adapted from Barberà et al.’s (2016) cross-national study of teachers’ perceptions of online learning success. The open-ended questions were developed based on the literature focused on online environments and taking into consideration the ongoing ERT. The statements and questions explored teachers’ i) views and attitudes towards online teaching, ii) curriculum planning and implementation, iii) assessment and student outcomes, iv) successes and challenges, v) support during transition to online teaching, and vi) recommendations for enhancing the quality of online teaching experiences for teachers and students alike.

In this study, teachers’ reflection on practice to evaluate the quality of teaching and learning experiences in online environments during ERT is warranted. Hodges and Fowler (2020) maintain that teachers’ reflections can lead to better teaching practices and better preparation for instructional situations such as ERT. Reflection can be defined as the careful examination of ideas through ongoing cycles of expression and re-evaluation in order to create new insight (Marshall, 2019, as cited in Hodges & Fowler, 2020). Both components (questionnaire statements and open-ended questions) required teachers to reflect on their practice. In this paper, data analysis and findings related to ii) curriculum planning and implementation and iii) assessment and student outcomes are reported.

### Data analysis

Initially the authors planned to collect data from 100 science/STEM teachers. This paper is based on data collected from 75 participants as data saturation occurred in the qualitative data, with similar trends highlighted in the quantitative data. Quantitative data analysis was conducted in MS Excel and SPSS. Descriptive statistics were performed in addition to various statistical tests to investigate the relationship or the correlation between various factors. Specifically, the Spearman correlation test (Connolly, 2007) was performed to explore the relationship between various demographic data.

**Fig. 2:**
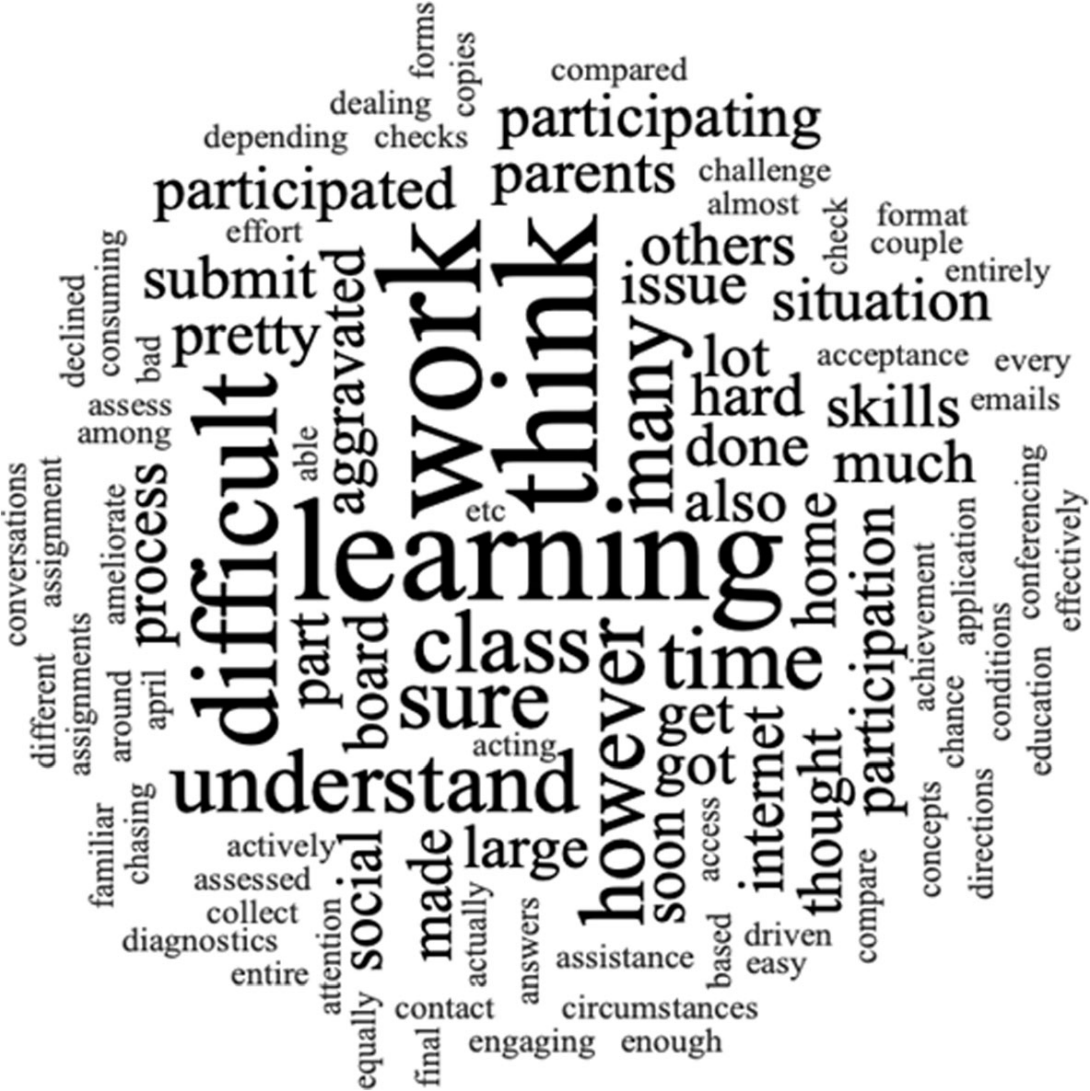
A sample word cloud generated in NVivo 12

Qualitative data from open-ended responses were analyzed through an interpretational analysis framework, using NVivo 12 data analysis software and executed through the process of thematic coding and constant comparative method (Stake, 2020). Participants’ reflections were inputted directly into NVivo 12, and emerging codes were generated via word clouds illustrating their frequency based on the size of the font (word frequency query) (see Fig. 2). These codes were then explored and interpreted to seek context as some words carry equal or similar meaning. Thereafter, similar codes were combined into themes. Thematic coding was performed independently by both authors to enhance the trustworthiness and the consistency of the analysis. Thereafter, the authors met and discussed the coding results and clarified discrepancies.

## Results and discussion

In the following sections, findings are presented based on the research questions focusing on digital resources, curriculum development, pedagogical approaches, assessment strategies, and student outcomes.

### What digital tools and resources were teachers using in an online environment?

Teachers used a variety of LMSs or platforms in their online teaching. An LMS is a software application that provides instructors and students with an interface to optimize online learning (Smith et al., 2018). Teachers used Brightspace/D2L, social media (e.g., Instagram, YouTube, Twitter, Facebook), video-conferencing tools (e.g., Zoom, Skype), online collaboration tools (e.g., Microsoft Teams and Google Classroom), as well as other platforms such as Classdojo (www.classdojo.com), Seesaw (https://web.seesaw.me/), Schoology (www.schoology.com), Edmodo (https://new.edmodo.com/), and Freshgrade (https://freshgrade.com/). The main decisive factors for choice of platform(s) were user-friendliness (34%), school board choice (27%), and interactivity (19%). Only 11% of the teachers stated that their choice of LMS was dictated by availability of features that specifically promote learning. The features sought by teachers included: enabling smooth video calling; providing a similar-to-classroom experience such as the ability for students to “raise their hand”, muting participants, and teacher control; pre-existing familiarity of students and parents with the LMS software; ability to organize all teaching and student assignments in one place; ease of creating and evaluating assessments; messaging and chatting options; and ability to customize. Finally, 9 % of respondents mentioned other reasons for LMS selection, such as previous usage by the teachers; security and privacy; login tracking; and ability to transfer content from one platform to another. Teachers’ choice based on the user-friendliness of the LMS is reflected in Darby’s recommendation (2019) that an LMS must be intuitively organized so that students find the content and activities easily. It should also show the expectations clearly as online students typically work independently and asynchronously. Hence, students cannot ask for, or receive clarification in the moment they first encounter the assignment instructions.

### What strategies did teachers’ online curriculum development and implementation embrace?

**Fig. 3:**
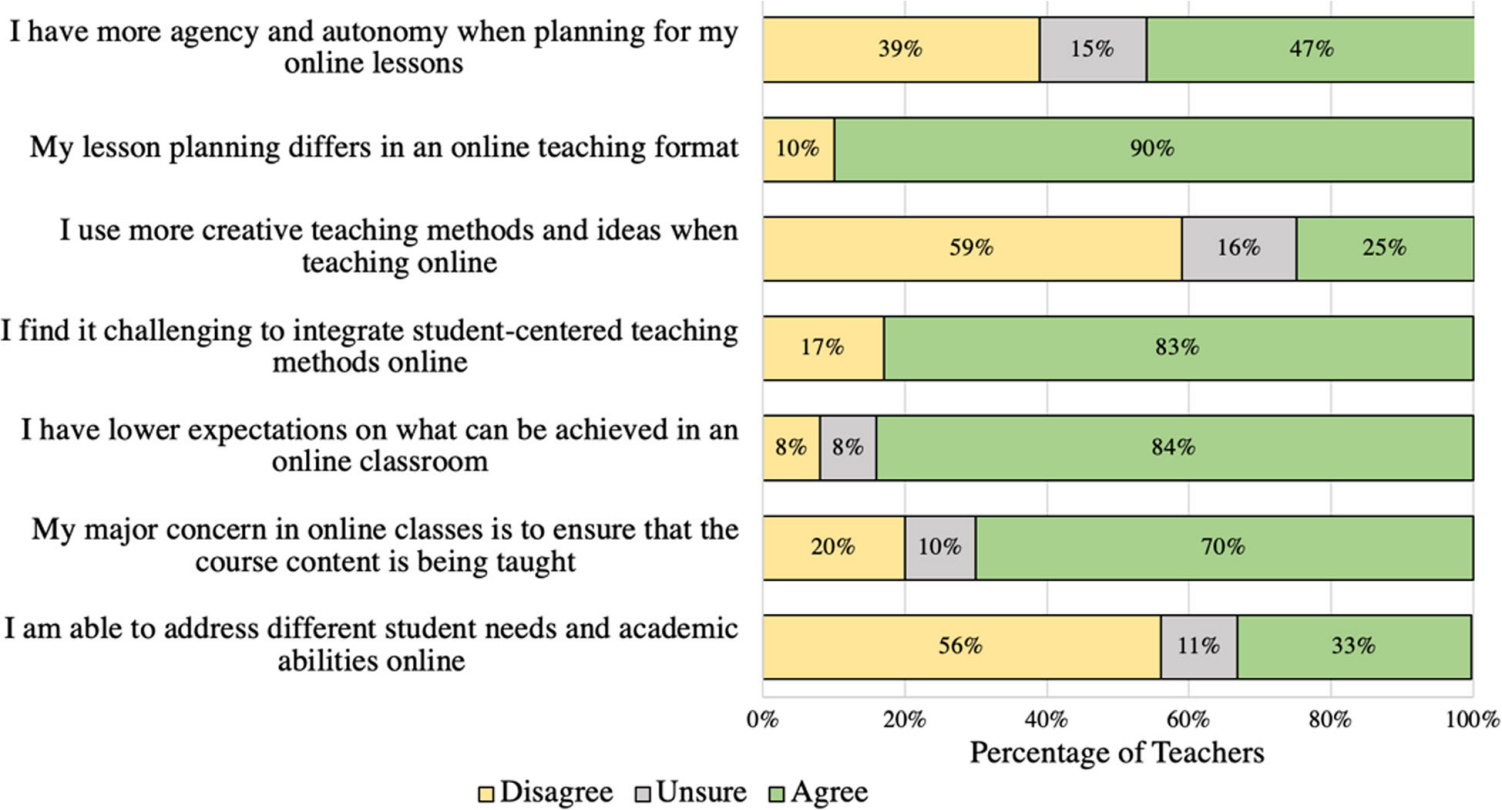
Teachers’ responses on their curriculum planning and implementation during ERT

Teachers reflected on their curriculum planning and implementation, as illustrated in Fig. 3. Although 47% agreed they had more autonomy in pedagogical choice, the implemented strategies appear to be less creative and less student-centered, thus failing to embrace pedagogy such as group discussions, online activities and simulations, virtual labs, and inquiry-based activities.

Ninety percent of respondents indicated that lesson planning for online implementation differed from traditional classroom teaching, with 59% admitting that they felt their online teaching methods were less creative compared to in-person classroom pedagogy. Eighty-three percent of responding teachers concurred with the statement “it is challenging to implement student-centered techniques in an online setting.” Reflecting these concerns is the observation that 84% of teachers indicated they had lower expectations about what can be achieved in terms of curriculum coverage and teaching methods during ERT, while 70% of teachers reported that they were more content-oriented rather than teaching-strategy oriented.

One can conclude that teachers had to prioritize content delivery at the expense of student-centered and creative teaching strategies. This is further supported by their choice of adopted teaching strategies. Teachers reported using self-directed learning in which they assigned specific readings or tasks for students to perform independently (23%); pre-recorded videos explaining the content (19%); multimedia including virtual labs and online simulations (18%), synchronous or live/direct teaching (15%); content creation tools such as video-making or presentations (14%); and other teaching methods (11%) such as online discussion tools for posting messages/questions or responding to a message or request after reading a chapter or completing a learning module. The aforementioned strategies for exchange of information and ideas occur asynchronously in most online teaching environments (Smith et al., 2018). Moreover, for the purpose of synchronous meetings, a few teachers printed and delivered materials on a weekly basis to students’ homes. This speaks to teachers’ commitment to supporting ongoing learning for their students, especially students with limited access to resources. Teachers also relied on a wide range of online resources to support teaching and learning such as YouTube, Khan Academy, online applications and websites (e.g., Gizmos, Edpuzzle, TVO, PhET interactives, Desmos, Classkick, Kahoot, Wizers, ArcGIS), digital textbooks, and teachers’ personal websites.

Therefore, based on teachers’ self- reporting one can infer that the quality of teaching and learning was compromised in favor of content delivery in the ETR context. Teachers were not able to create communities of inquiry (Barril, 2018; Garrison & Arbaugh, 2007) nor utilize student-centered innovative teaching methods (Dipietro, 2010). Dewey (1906) promoted student-centered innovative teaching and learning approaches and warned educators about three “red lines”: i) lack of connection with what the child feels and loves, ii) lack of motivation needed for learning, and iii) presenting ready-made curriculum. It is evident that the online teaching practices during the ERT paralleled Dewey’s red lines, as teachers presented content-heavy curriculum and relied upon traditional teaching practices (Baran et al., 2011). These results also reflect teachers’ self-efficacy (Bandura, 1995) and TPACK (Koehler & Mishra, 2009) framework, and reiterate the claims that online teaching requires the intersection of the knowledge constructs of TPACK, as well as teachers’ perceptions, beliefs and self-efficacy around teaching in said environments (Dipietro, 2010). In this study, teachers were not adequately prepared in terms of integrating TPACK in their practice and faced many challenges (DeCoito & Estaiteyeh, in press) that severely impacted their digital self-efficacy. This speaks to the importance and relevance of the CIPP model in terms of integrating quality processes (curriculum and digital resources, teaching and assessment using digital tools) to ensure expectations and outcomes are realized (Stufflebeam, 2007).

Teachers recognized the shortcomings in trying to implement traditional teaching methods during ERT. For example, 56% of respondents indicated they were unable to differentiate instruction and accommodate all students in an online environment. This finding parallels those from studies highlighting challenges related to differentiated instruction in online environments (Cook & Steinert, 2013; Dipietro et al., 2008; Smith et al., 2018). This is critical as it reinforces the documented concerns around equity, diversity and inclusion of disadvantaged students and underprivileged communities in online classes (Lao & Gonzales, 2005; Rohleder et al., 2008; Stelitano et al., 2020). This also counteracts one of Dewey’s (1906) foundations of student-centered learning – the well-knowing of students and personalizing the teaching to match their interests, capacities, attitudes, and experiences.

### What models of student assessment did teachers implement online?

Teachers reported the use of a variety of assessment strategies including online quizzes and tests (71%), homework (35%), projects (35%), and labs or simulations accompanied by worksheets or questions (25%) (Table 2). Moreover, 18% of teachers reported utilizing multimodal evidence of learning, as chosen by their students (voice or video recordings, written themes, images including digital collages and hand-drawn scanned artwork, digital notebooks). An additional 18% of teachers used personalized feedback and rubrics.

**Table 2:** Teachers’ assessment strategies in an ERT context

Modes of Assessment	Frequency (%)
Online quizzes and tests	71
Homework	35
Projects (individual or group)	35
Labs or simulations or audio-visual with worksheet/follow-up questions	25
Evidence of learning in the format of their choice	18
Teacher feedback and rubrics	18
No assessments were allowed	12
Participation/ discussions	9
Self-assessment	4

*Note:* Every teacher described several assessment strategies they had implemented

Based on these findings, the authors conclude that teachers in this study were relying on traditional assessments in favor of more creative ones, with quizzes and tests being the most common assessment tool. The use of traditional assessment methods parallel McVey’s (2016) study findings in which pre-service teachers cautiously made use of more traditional tools such as quizzes and reports in an online teaching context. Given the overwhelming ERT context, teachers in our study were challenged in the ‘new’ learning environment due to insufficient prior exposure and training, and as such teachers lacked the time and skills necessary to implement more authentic and student-centered assessments. This further highlight concerns about online assessment (Amasha et al., 2018; Eichler & Peeples, 2013; Espasa & Meneses, 2010; Tinoca & Oliveira, 2013), and confirm findings by Anderson (1998) linking the choice of assessment tools to the use of student-centered teaching methods.

As shown in Table 3, 82% of teachers stated they promptly returned all assignments to their students and provided individualized useful feedback. This suggests that teachers were focused on student growth. Despite the inclusion of some creative assessment strategies, online delivery of traditional assessment methods (71%, Table 2) and numerical grading (56%, Table 3) were the most common means of evaluating student learning. As a result, this may have influenced the authenticity of the assessment tools and posed several challenges in the ERT environment.

**Table 3:** Teachers’ responses on their assessment strategies during ERT

Statement	Never	Sometimes	About half the time	Most of the time	Always
I returned all assignments promptly.	2.78%	6.94%	8.33%	44.44%	37.50%
I provided individualized and useful feedback/guidance that met learners’ needs.	1.39%	4.17%	12.50%	37.50%	44.44%
I used numerical grading in my assessments.	34.72%	9.72%	5.56%	22.22%	27.78%

#### Reflecting on assessment

Teachers reflected negatively on their assessment strategies. Seventy-six percent of respondents viewed their assessment strategies as ineffective. In contrast, 15% described their assessment strategies as effective, while 9 % were unsure about the efficacy of their assessment tools. The ineffectiveness of assessments directly relates to their level of authenticity, which is defined by the degree of resemblance to the criterion situation (Gulikers et al., 2004). Frey (2018) describes authentic assessments as those involving the student in cognitively complex and interesting situations, thus allowing students to demonstrate their competencies to engage in further knowledge construction. Authentic assessment also provides multiple ways for students to demonstrate their learnings. Hence, it is assessment “for learning”, not only “of learning”.

**Fig. 4:**
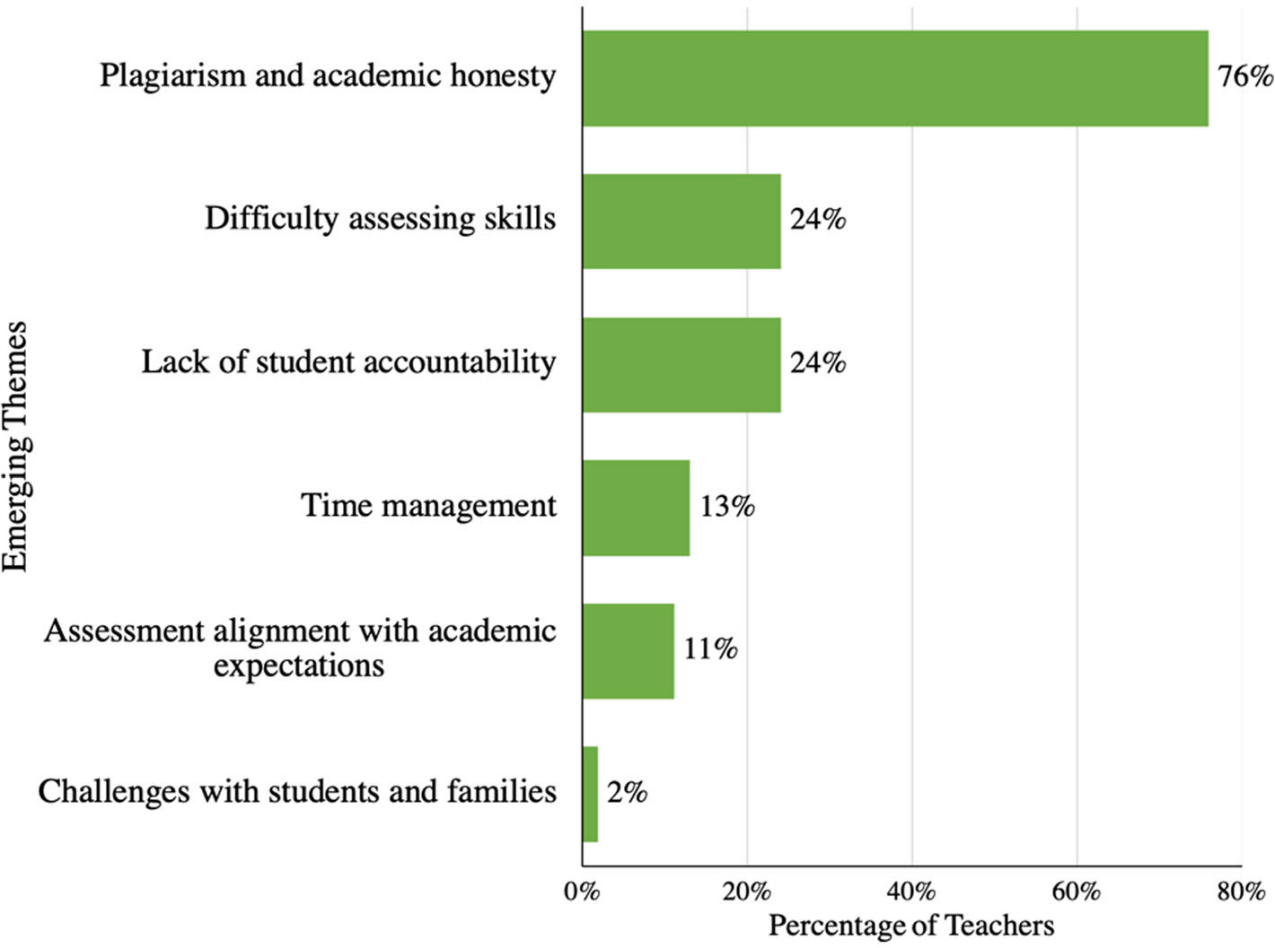
Themes related to reasons for ineffective online assessment strategies

Study participants shared in their reflections why they perceived their online assessment strategies as ineffective. Additional analysis of teacher responses revealed the following themes: plagiarism/academic dishonesty (76%); assessing skills (especially higher order thinking skills) (24%); lack of student accountability (24%); time management (13%); assessment alignment with academic expectations (11%); and challenges with students and families (2%) (Fig. 4).

**Fig. 5:**
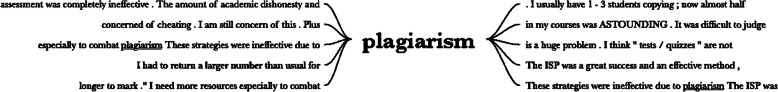
NVivo 12 generated word tree highlighting teachers’ reflections on *plagiarism*

Plagiarism and academic dishonesty had the greatest impact on teachers’ perceptions of ineffective online assessment. Teachers identified multiple forms of student plagiarism including the use of search engines as open books during tests, family members completing and submitting work on behalf of the students, and sharing answers between peers (Fig. 5).

Most teachers were frustrated by their limited ability to control or restrict plagiarism and deemed this as the main challenge to the authenticity of assessments. Teachers commented:

*The amount of plagiarism in my courses was astounding.* (Biology and chemistry teacher)

*We had a lot of problems with this because the students would blatantly cheat from each other. And also (sic) copy solutions from online calculators which will show steps. All-in-all this method of assessment was not very successful.* (Secondary math teacher)

*There could be no certainty that their parents/siblings weren’t completing their work.* (Elementary science and technology teacher)

*It was next to impossible to ensure students are (sic) truly doing their own work without outside assistance. Written reports were more effective, especially with originality reports generated by Google Classroom/other "Turn-it-in" styles programs.* (Biology and math teacher)

Challenges related to cheating and plagiarism in online assessments are well-documented. According to Palloff and Pratt (2007), proactive measures such as properly educating students and developing the notion of community of learners to mitigate the abundance of such behavior are recommended. This also reiterates the importance of teacher preparation in terms of dealing with plagiarism (Leire et al., 2016).

Additionally, teachers expressed challenges assessing their students in general, with several articulating specific challenges with assessing higher order thinking skills, lab-based technique skills, and manipulative skills. Some teachers noted that providing online feedback was not as effective when compared to in-class feedback. Teachers commented:

*The Gizmos and virtual labs are a good online tool (sic) BUT students miss the practical skills involved in labs and collaboration with other students.* (Secondary biology teacher)

*I felt it was very knowledge/ understanding heavy compared to inquiry process and investigation. Skills were mostly textbook based compared to hands on problem solving.* (Secondary math teacher)

The literature documents several assessment strategies that can help teachers assess collaborative and higher order thinking skills (Hamann et al., 2016; Leire et al., 2016). Our findings highlight professional development opportunities to ensure that teachers are well-prepared to utilize a variety of assessment tools/strategies.

Several teachers commented on the lack of student accountability as one of the challenges when assessing online learning and attributed this to a lack of student engagement. Many teachers also related this to inequity amongst students. For example, disparities in terms of access to appropriate technology, digital competence, and parental support amongst vulnerable and disadvantaged students magnified the challenges. Teachers noted:

*Engagement was difficult as many students understood that in a way, this learning was optional since they achieved a passing grade prior to school closing.* (Computer studies teacher)

*Students complained about accessing all types of materials. The complaints were real, as many were not tech savvy, and had to be walked through the process.* (Chemistry and math teacher)

*For my students with special needs, our classes that are normally hands on science could not be done at home since some of them had no equipment, space, or someone at home to assist them with activities. I could not assess their skill development that I normally would evaluate in the course.* (Sciences teacher)

*Online assessment strategies were confounded by health/mental health factors related to the pandemic, issues of equity and accessibility of technology, and motivation.* (Health and physical education teacher)

Consequently, many teachers felt that online learning and assessment did not accurately reflect academic expectations. Teachers seemed unsure about whether their students really understood concepts or mastered skills, which also calls into question the authenticity of the assessment. Teachers said:

*(I was) not always sure if they get the concept taught.* (Chemistry teacher)

*Some students didn't submit these assignments and others copied from each other. Hence not all students were able to understand the concepts and master the required skills.* (Math and physics teacher)

*Although I did think students were learning and meeting some of the curriculum expectations of the course, I do not feel that the assessment strategies were very effective in evaluating students' understanding of concepts and mastery of skills. I really struggled to create valid evaluations since everything the students did was "open book". I think this is a mindset change that needs to be worked on with many STEM teachers.* (Secondary biology teacher)

Finally, it is important to note that 11% of teachers indicated that school board policy prohibited student assessment during ERT. Some of those teachers mentioned:

*We did not evaluate the online projects. Our marks were based on marks before March Break.* (Elementary math, health and physical education, and design and inquiry teacher)

*We were told not to assess our students in an online environment.* (Elementary math and science teacher)

*I did not assess my students in the online environment. Feedback was given for each task.* (Elementary science and technology teacher)

These responses highlight challenges and tensions around assessment practices in an ERT context. Some teachers linked assessment to grading and believed that their assessment was invalid or did not count since students were not graded. One of the participants did not consider the feedback they provided on tasks as an assessment strategy. Overall, teachers’ responses reflected a major gap in their understanding of assessing online learning (Espasa & Meneses, 2010; Tinoca & Oliveira, 2013). This gap can be addressed through professional development programs and high-quality resources reflecting authenticity in assessment strategies. Such programs should integrate TPACK (Koehler & Mishra, 2005) in order to model linkages between pedagogical approaches, content knowledge, and assessment in online environments.

#### Conditions for effective assessment

Fifteen percent of responding teachers reported on criteria for effective online assessment including record keeping tools; communication and feedback; assessment “for learning” through written reports and rubrics instead of tests; and extending time and pacing for students. Despite their satisfaction, responses were still coupled with challenges associated with providing effective online assessment. In utilizing the online learning platform, some teachers found the embedded rubrics, mark managing software, and plagiarism software to be helpful and expressed intentions to utilize these tools upon return to face-to-face instruction. Teachers commented:

*Online assessment was effective as it was based on rubrics.* (Elementary science and technology teacher)

*It was great actually. Students were able to get immediate feedback on whether they understood the material. These didn't count towards their grade - just self-assessment. But it also allowed me to gauge whether they were engaging with the material each day.* (Math teacher)

*Assessment strategies used are very effective. While the learning is very student driven, the outcomes could be assessed with a simple rubric and other specific diagnostics depending on the assignment.* (Earth and space sciences, environmental sciences, and computer studies teacher).

The effective use of online feedback and rubrics are emphasized in the literature (e.g., Dipietro, 2010). Online environments are conducive to these forms of assessment in terms of providing both teachers and students with valuable information about student progress.

Teachers expressed the need for communication in successful assessment, including providing written feedback that allows for more meaningful conversations, consistent and personal follow-up with students, and clear instructions. Teachers commented:

*I think they were pretty effective because I made sure to have a lot of contact with students - conferencing and small groups, emails, etc. to be sure they were learning*.

*My assessment strategies seemed very much for assessment FOR learning purposes only. It felt unfair to do much assessment of learning due to the platform being new to students.*

The latter comment, in addition to teacher responses on effective assessments, highlight adequate levels of understanding of assessment. This further emphasizes the importance of teacher collaboration and exchange through communities of practice (Wenger, 1998) that would engage teachers in rich and fruitful discussions about online assessment strategies. In an ERT context, technological, pedagogical, and social challenges experienced by teachers are numerous (Ferri et al., 2020), thus impeding their ongoing participation in communities of practice.

### What were the impact(s) of online teaching on student outcomes, as observed by teachers?

**Fig. 6:**
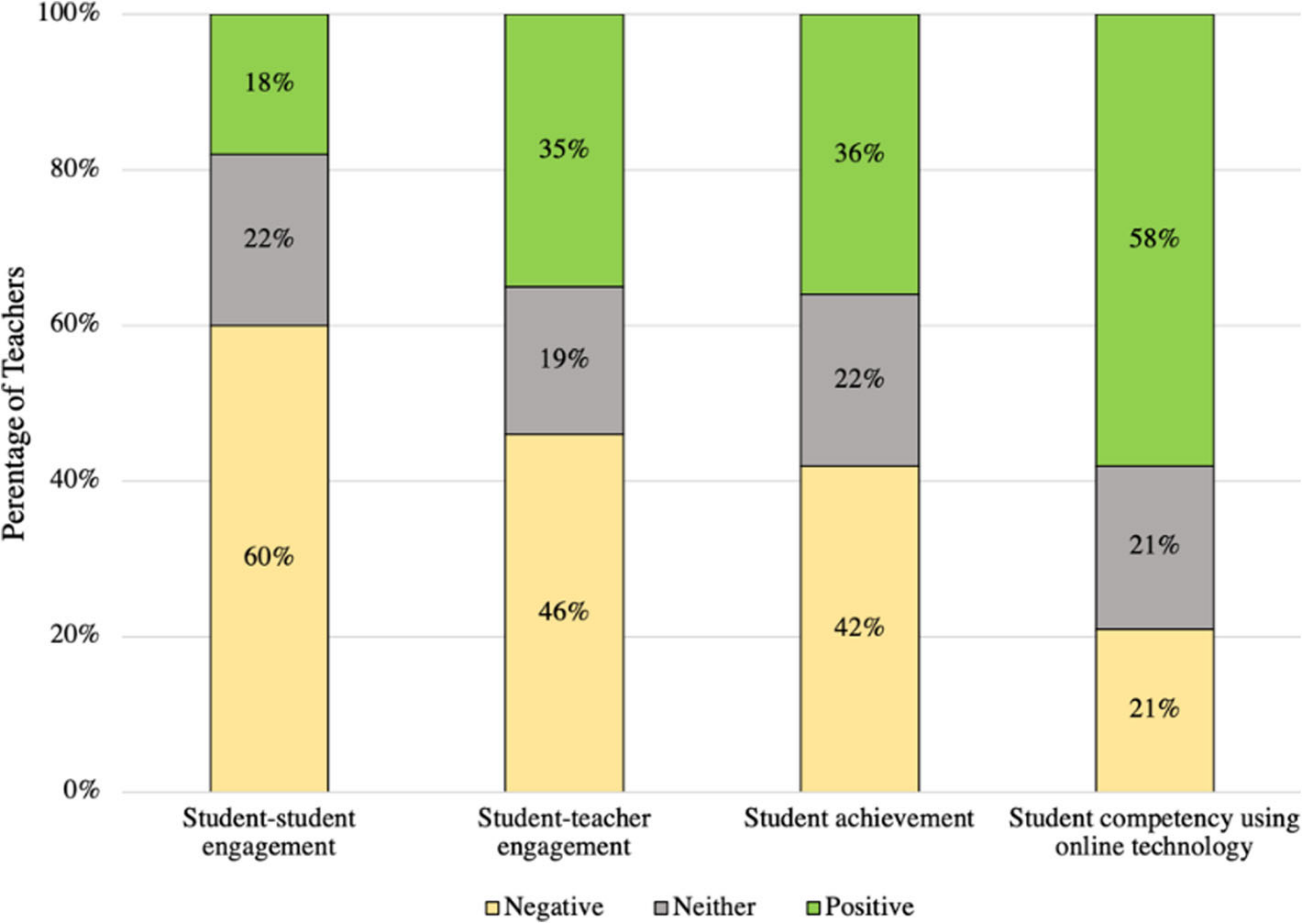
Teachers’ account of online teaching impact on student outcomes

The impact of online teaching on student outcomes was generally considered negative by most teachers (Fig. 6). The observed negative impact relates to student-student engagement (60%), student-teacher engagement (46%), and student achievement (42%). This reiterates the aforementioned challenges of online teaching that have been documented in the literature (Barril, 2018; Recker et al., 2013; Searls, 2012; Simonson et al., 2009; Tinoca & Oliveira, 2013; Zhang & Lin, 2020). On the other hand, 58% of teachers observed a positive impact on student competency in using online technology. Educators can capitalize on students’ digital competence to enhance their engagement and reflect academic gains in online environments. The current technological advancements, especially social media and communications can help mitigate these challenges. Research has documented that online digital resources can enrich the classrooms and improve student learning (Recker et al., 2013).

The impact of online teaching on student outcomes is inconclusive. While some studies have highlighted the positive impact of Internet-supported learning on students in terms of grade achievement, motivation, participation, and satisfaction (Amasha et al., 2018; Bekele & Menchaca, 2008; Dumford & Miller, 2018), several studies highlight challenges related to learners’ motivation and engagement (Cook & Steinert, 2013; J. Davis et al., 2007; Lao & Gonzales, 2005; Leire et al., 2016; Saadé et al., 2007; Zhang & Lin, 2020). It is also important to note the context of these studies. Teachers and students in our study were faced with many challenges related to ERT that negatively affected student outcomes. These findings reiterate the importance of aligning elements of the CIPP model during ERT, specifically context, process, and products, and recognizing the interconnectedness of these elements (Stufflebeam, 2007). Furthermore, we need to take into consideration other factors that affected students’ motivation and accountability including their mental well-being and interest in learning, in general, during this unprecedented time. This complicates the analysis but does not negate the fact that students and teachers were facing several challenges throughout the process.

## Conclusions

This study sought to explore science/STEM teachers’ curriculum and assessment practices during ERT. Findings suggest that a model, such as CIPP, is essential for organizing online teaching and learning. For example, despite teachers organizing online lessons during ERT, gaps were identified in teachers’ TPACK frameworks and self-efficacy. These in turn impacted teachers’ curriculum development, pedagogy, and assessment practices. In terms of teaching strategies and curriculum implementation, results of this study indicate that teachers prioritized teaching subject content knowledge at the expense of implementing creative and student-centered pedagogy. These findings directly correlate with teachers’ TPACK, as despite 86% of teachers self-reporting high competency scores for online technology teaching, they faced difficulties combining their technological skills, pedagogical skills, and content knowledge to ensure a rich online teaching/learning experience. This is linked to teachers’ self-efficacy – with low self-efficacy negatively affecting attitudes toward online teaching (DeCoito & Estaiteyeh, in press), and subsequently, pedagogical practices related to online contexts.

Online assessment techniques, which generally did not include many creative tools, were viewed by teachers as mostly ineffective and unauthentic. For example, 76% of teachers did not view their assessments as effective. Furthermore, most teachers did not view the impact of online teaching on student achievement and engagement positively. These findings directly relate to teachers’ self-efficacy and the integration of the TPACK framework in addressing elements of CIPP, specifically process and outcomes (Stufflebeam, 2007).

In this study, science/STEM teachers’ online pedagogical and assessment practices during the COVID-19 pandemic warrant further investigation. Teachers expressed several challenges impacting their attitudes toward online teaching, which in turn negatively affected their pedagogical practices. These challenges include the lack of high-quality resources related to teaching and assessment, and lack of training; student equity issues in terms of access, technological skills, support, and special-needs students; and teacher time constraints (DeCoito & Estaiteyeh, in press). These challenges negatively impacted teacher reported student outcomes, given that an increase in teachers’ outcome expectancies reflect a greater belief in their students’ abilities to succeed (Riggs & Enochs, 1990; Teo, 2009).

Our findings add to the literature on ERT, enhancing teacher self-efficacy and TPACK understandings. Many of the challenges identified warrant ongoing professional development opportunities – collaborative work groups, community building and communities of practice – to transform teachers’ practices through social knowledge construction (King, 2002). For example, teachers need to be prepared and supported for online teaching to become well versed in the relevant instructional online pedagogies (Baran et al., 2011). Furthermore, teachers must carefully balance between the time spent on pedagogical practices and that spent on managerial ones (Zhang & Lin, 2020) in online contexts. Finally, teachers should engage in professional development around technology through workshops and training sessions (Smith et al., 2018) to improve their self-efficacy and TPACK integration. We believe that this research is instrumental for developing a framework that is appropriate to evaluate ERT practices in science/STEM education.

One key finding that warrants extensive investigation beyond the scope of this paper is teachers’ account of challenges providing equitable and inclusive online teaching. Teachers reported the lack of equity and inclusion of disadvantaged students or underprivileged communities in online classes, similar to findings of other studies (Ferri et al., 2020; Stelitano et al., 2020). This is an ongoing challenge in education, irrespective of the pandemic, however, it was exacerbated in an ERT context.

Regardless of the status of the COVID-19 pandemic or potential pandemics in the future, online teaching is a promising endeavor in a growing digital world. Thus, K-12 teachers need to be equipped with the required digital literacy skills and be prepared to teach in environments where students have a great interest and where they can capitalize on their proficiency to excel academically (DeCoito & Richardson, 2018b; Ng, 2013).

## Implications and future research

This research is instrumental for providing a landscape of challenges, successes, gaps and barriers encountered by teachers and students as they migrated to online teaching during a global pandemic. It will advance knowledge about online teaching in K-12 settings. Moreover, it will inform policy makers, administrators, and curriculum designers about successes and challenges associated with ERT. Our hope is that it provides teachers with an opportunity to reflect on and assess their current practices and explore other teachers’ practices in science/STEM education. It beckons the education community to explore and innovate various ways to enhance student-student engagement and student-teacher engagement online; develop authentic digital assessment tools; and promote inclusive and relevant curriculum and pedagogy.

Future research may explore ERT nationally and internationally with a goal of developing new frameworks for addressing online teaching and assessment practices in science/STEM education. Future research should focus on online observations of teacher practice, as well as explore the effectiveness of professional development programs on pre-service and in-service teachers’ pedagogical practices. Finally, student outcomes should be explored in depth (through interviews, observations, and coursework analysis) to gain a comprehensive understanding of the implications of ERT on science/STEM teachers and students.

## Data Availability

Raw data is available for transparency purposes.

## Abbreviations

STEM: Science, technology, engineering and mathematics
ERT: Emergency remote teaching
CIPP: Context, input, process and product
TPACK: Technological, pedagogical, and content knowledge
LMS: Learning management system
